# Dissecting the link between the enzymatic activity and the SaPI inducing capacity of the phage 80α dUTPase

**DOI:** 10.1038/s41598-017-11234-9

**Published:** 2017-09-11

**Authors:** Christian Alite, Suzanne Humphrey, Jordi Donderis, Elisa Maiques, J. Rafael Ciges-Tomas, José R. Penadés, Alberto Marina

**Affiliations:** 1 Instituto de Biomedicina de Valencia (IBV-CSIC) and CIBER de Enfermedades Raras (CIBERER), Jaume Roig 11, 46010 Valencia, Spain; 2 Institute of Infection, Immunity and Inflammation, College of Medical, Veterinary and Life Sciences, University of Glasgow, Glasgow, G12 8TA UK; 3 Present Address: Departamento de Ciencias Biomédicas, Universidad CEU Cardenal Herrera, 46115 Alfara del Patriarca, Valencia, Spain

## Abstract

The trimeric staphylococcal phage-encoded dUTPases (Duts) are signalling molecules that induce the cycle of some Staphylococcal pathogenicity islands (SaPIs) by binding to the SaPI-encoded Stl repressor. To perform this regulatory role, these Duts require an extra motif VI, as well as the Dut conserved motifs IV and V. While the *apo* form of Dut is required for the interaction with the Stl repressor, usually only those Duts with normal enzymatic activity can induce the SaPI cycle. To understand the link between the enzymatic activities and inducing capacities of the Dut protein, we analysed the structural, biochemical and physiological characteristics of the Dut80α D95E mutant, which loses the SaPI cycle induction capacity despite retaining enzymatic activity. Asp95 is located at the threefold central channel of the trimeric Dut where it chelates a divalent ion. Here, using state-of-the-art techniques, we demonstrate that D95E mutation has an epistatic effect on the motifs involved in Stl binding. Thus, ion binding in the central channel correlates with the capacity of motif V to twist and order in the SaPI-inducing disposition, while the tip of motif VI is disturbed. These alterations in turn reduce the affinity for the Stl repressor and the capacity to induce the SaPI cycle.

## Introduction

*Staphylococcus aureus* pathogenicity islands (SaPIs) are a group of related ≈15 kb mobile genetic elements that commonly carry genes for virulence factors and are responsible for their dissemination^1^. In addition to being a reservoir for virulence factors, another interesting aspect of SaPI biology is related to their mobility. SaPIs have an intimate relationship with certain temperate phages which induce them to excise and replicate^2^. Following replication, SaPI DNA is packaged into small viral-encoded icosahedric proheads that accommodate their smaller genomes^3–5^. These particles enable very high frequency transfer, not only among staphylococci, but also to other bacterial genera^6–8^, where their integration into the bacterial chromosome is mediated by the activity of the SaPI-encoded integrase^3, 8, 9^, enabling them to be stably maintained. Maintenance of SaPI in the integrated state depends upon expression of a SaPI-encoded repressor, Stl^10^. In contrast to most of the phage-encoded repressors, Stl is not degraded by RecA*, and consequently, SaPI induction does not depend directly on the SOS response. Instead, SaPI derepression is facilitated by phage-encoded moonlighting proteins, which bind to the Stl repressor, activating the SaPI cycle^1^.

Both the trimeric and the dimeric phage-encoded dUTPase proteins (Dut) act as the de-repressor protein for a subset of SaPIs, including SaPIbov1, SaPIbov5 and SaPIov1^2^. Interestingly, a comparison of the trimeric Dut sequences from various staphylococcal phages revealed high sequence similarity, except for a non-conserved central region, defined as motif VI. This motif is highly divergent among *S. aureus* phage enzymes^2^ but importantly is not required for enzymatic activity^11, 12^ and is absent in some functionally-related Duts from other species. However, our results revealed that motif VI plays an essential role in the interaction with the SaPI-encoded Stl repressor^2, 12, 13^. Although necessary, motif VI is not sufficient to induce the SaPI cycle^13^. Unexpectedly, the C-terminal P-loop motif V as well as the motif IV, two motifs conserved in all the characterised trimeric Duts (from phage to human), also play a key role in mediating derepression^12, 13^.

In the original work that identified the trimeric Duts as the inducing proteins for SaPIbov1, we proposed that the dUTPase activity was not required to derepress the SaPI cycle^2^. This was supported by two complementary results: firstly, we obtained a phage 80α carrying the D95E mutation in the Dut protein. While this phage was incapable of inducing the SaPIbov1 cycle, the mutant Dut protein retained its enzymatic activity when overexpressed in *E. coli* ^2^. Secondly, when analysing the phage ϕ11 Dut protein, we observed that overexpression of the catalytically inactive ϕ11 Dut D81A mutant induced SaPIbov1^2^.

However, in a subsequent study, while analysing Dut mutants from different phages, we implicated the dUTPase activity in the Dut-mediated induction of the SaPI cycle. Specifically, we observed that for the catalytically inactive ϕ11 or ϕ71 Dut D81A mutants, the affinity of these enzymes for the Stl repressor was lower than that observed for the wt proteins^13^. Moreover, in relation to phage 80α Dut, the analysis of different mutants mapping the active site of the enzyme revealed that the enzymatic activity was absolutely required to induce the SaPI cycle. Thus, all of the enzymatically inactive 80α Dut mutants analysed were unable to induce the SaPI cycle^13^. Since the three-dimensional structures revealed that the conserved motif V was disordered in all these inactive mutants, our initial results suggested that the correct ordering of the 80α Dut motif V over the active centre of the enzyme is an essential requisite for initiating SaPI depression^13^. However, this assumption was incorrect, and we and others have recently demonstrated that the dUTP molecule blocks the Stl:Dut interaction^12, 14^, allowing us to propose a consensus model for Stl-Dut interaction where the *apo* form of the Duts provides the competent conformation for interacting with the Stl repressor. In this conformation motifs IV and VI provide the recognition and binding site for the Stl repressor, meanwhile the highly flexible motif V stabilizes the Stl-Dut complex once the repressor binds to the Dut. Conversely, in the dUTP-bound form, the highly flexible motif V folds over the active center, covering the Stl binding site provided by motifs IV and VI and precluding Stl-binding. Based on the aforementioned model, the phenotype observed for the previously characterized 80α D95E Dut mutant, which is enzymatically active but does not induce the island, is difficult to explain. Interestingly, an Aspartic residue at the position of 80α Dut Asp95 is highly conserved in all *S. aureus* phage-encoded trimeric Duts^11, 12^. The three-dimensional structures of Duts from phages 80α and ϕ11 have shown that this residue maps to the N-terminal beginning of motif VI facing the characteristic threefold central channel of trimeric Duts. In the trimer, the three Asp95, one from each subunit, participate in the coordination of one divalent metal ion (it has been modelled as Ni^2+^ or Mg^2+^ for 80α and Mg^2+^ for ϕ11) that occupies the central part of the channel, stabilizing motifs VI and/or the trimer^13^. Stabilization by interaction across the threefold central channel is a characteristic of trimeric Duts, although the nature of these interactions differs among Dut groups, being more hydrophobic in prokaryotes than in eukaryotes, and including in the latter group the presence of ions in the channel^15^. Recently, the conformational restriction of the central channel has been linked to the exquisite specificity for dUTP displayed by the Duts with respect to other structurally related members of the dUTPase superfamily, such as the bifunctional dCTP deaminase-dUTPase (DCD-DUT)^15^. Altogether, a steady relationship between Dut trimer stability, catalytic activity, nucleotide binding and selectivity with SaPI induction seems to be occurring in a manner dependent on the conserved Asp residue, which is present in all of the *S. aureus* phage-encoded Duts.

We consider this relationship an important question, since our current working hypothesis is that the phage Dut proteins are signalling molecules that interact with cellular partners using a mechanism similar to that used during derepression of the SaPIs^16^. Our thoughts are based on the fact that the SaPIs severely affect phage reproduction, indicating that the Stl-Dut interaction is detrimental for the phage^17^. However, we hypothesise that phages cannot escape from this interaction because the Stl repressor has mimicked the structure of one of the cellular/phage partners with which the Dut interacts in order to perform its regulatory role. Consequently, we expect that by deciphering the mechanism by which the Dut interacts with the Stl protein, we will be able to unravel the mechanism by which the Duts perform their regulatory functions. Taking this into account, here we provide insights into the mystery related to the 80α Dut D95E mutant.

## Results

### SaPI induction capacities of the phage 80α-encoded Dut D95E

We had previously reported that the phage 80α Dut (Dut80α) D95E mutant was unable to induce the SaPIbov1 cycle^2^. However, the molecular basis for this observation was not further analysed. Since the Dut80α D95E mutant retained the capacity to hydrolyze dUTP when overexpressed in *E. coli*, and since previous studies have demonstrated a link between enzymatic activity and the capacity to induce the SaPI cycle^13^, we initially hypothesised that the stability of this mutant protein was reduced *in vivo*. To test this, the gene encoding the Dut80α D95E protein was cloned in expression vector pCN51^18^. In this plasmid, the cloned *dut* gene is expressed under inducing conditions from the *P*cad promoter present in the plasmid^18^. Moreover, this plasmid expresses a 3× Flag-tagged version of the Dut80α D95E mutant which allows us to monitor the stability of the mutant protein when expressed in *S. aureus* cells. Note that previous studies have demonstrated that the 3× Flag does not affect either the inducing or the enzymatic activities of the Dut proteins^13, 17^.

**Figure 1:**
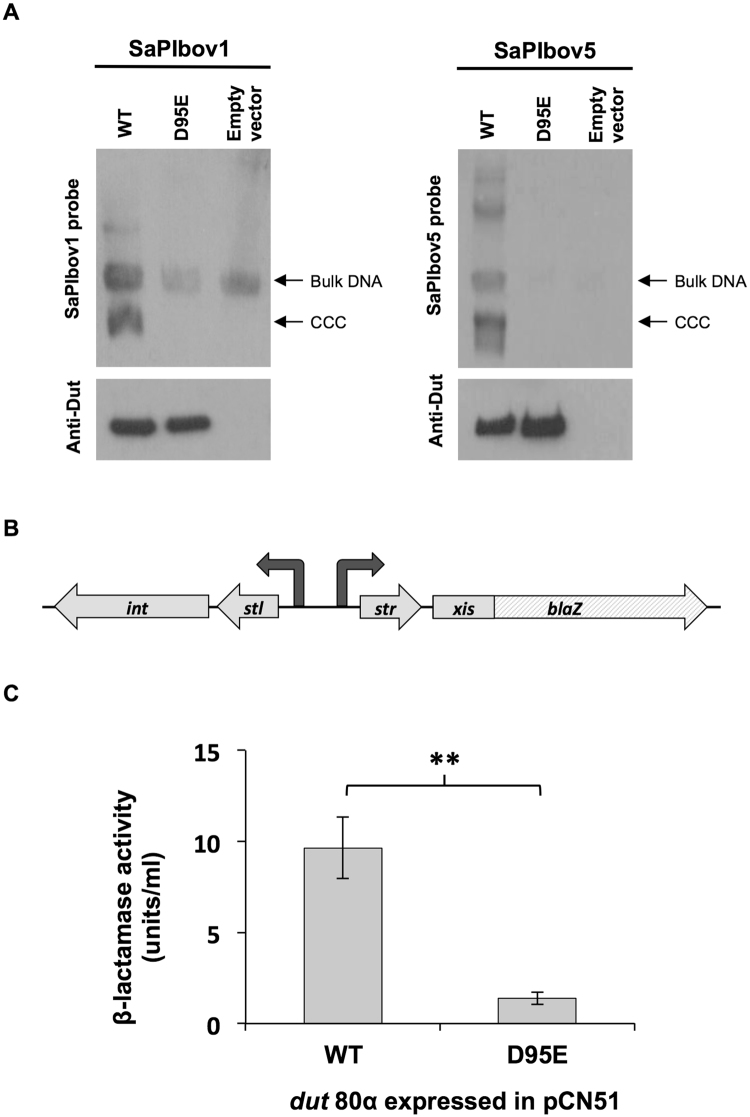
Induction of SaPIbov1 and SaPIbov5 by Dut80α D95E. (**A**) A non-lysogenic derivative of strain RN4220 carrying SaPIbov1 or SaPIbov5 was complemented with plasmids expressing 3× Flag-tagged Dut80α proteins (wt or D95E), or with empty plasmid pCN51. One millilitre of each culture (optical density (OD_540_ = 0.3) was collected 3 h after treatment with 5 μM CdCl_2_ and used to prepare standard minilysates, which were resolved on a 0.7% agarose gel, Southern blotted and probed for SaPI DNA. In these experiments, because no helper phage is present, the excised SaPI DNA appears as covalently closed circular molecules (CCC) rather than the linear monomers that are seen following helper-phage-mediated induction and packaging. The lower panel is a western blot probed with antibody to the Flag tag carried by the proteins. Only the lines of interest are shown from full-length blots that are included in Supplementary Fig. S3. (**B**) Schematic representation of the *bla*Z transcriptional fusion generated in plasmid pJP674. (**C**) Derepression of *str* transcription by *dut* expression. Strains containing pJP674- and pCN51-derivative plasmids expressing Dut80α or Dut80α D95E were assayed for β-lactamase activity in the absence of or 5 h after induction with 5 μM CdCl_2_. Samples were normalized for total cell mass. Data are from an experiment in triplicate. Error bars represent s.d. Asterisks denote statistical significance at *p* < 0.01 using an unpaired Student’s t-test.

Expression of the cloned gene in SaPIbov1- or SaPIbov5-containing strains confirmed that the Dut80α D95E mutant was incapable of inducing the SaPI cycle, even when overexpressed (Fig. 1A). As the Dut80α D95E protein level produced from this construct was comparable to that obtained when the plasmid encoding the wt protein was analysed (Fig. 1A), this result indicates that the D95E mutation does not affect the stability of the Dut mutant protein.

Further, to complete the characterisation of the *in vivo* inducing capacities of the Dut80α D95E mutant, we used plasmid pJP674, which carries a β-lactamase reporter gene fused to *xis*, downstream of *str* and the Stl-repressed *str* promoter, as well as encoding Stl (see Fig. 1B). A strain carrying this plasmid does not express the β-lactamase reporter gene because of the Stl-mediated repression of the reporter gene. pCN51 plasmids expressing the Dut80α D95E or the wt proteins were introduced into strain RN4220 carrying plasmid pJP674 and the capacity of the different proteins to induce the expression of the β-lactamase reporter gene was tested in the presence or absence of an inducing concentration of CdCl_2_. As expected, expression of the Dut80α D95E mutant failed to induce the expression of the β-lactamase reporter gene, although there was full derepression by the wt Dut under these conditions (Fig. 1C).

Finally, we introduced the SaPIbov1 and SaPIbov5 islands into strain JP4480, which carries the 80α prophage mutant in the *dut* gene. SaPIbov1 carries a *tet*M marker inserted in the *tst* gene, while SaPIbov5 carries a *tet*M marker in the *vwb* gene. These markers facilitate the transfer studies. Once these strains were generated, the pCN51 derivative plasmids expressing the Dut80α wt or the Dut80α D95E proteins, or the empty pCN51 plasmid, were introduced in the aforementioned strains. Then, the cloned Dut proteins were expressed, the cycle of the phage 80α Δ*dut* was SOS (mitomycin C) induced and the transfer of the islands was analysed (Table 1). In this type of experiment, if the cloned *dut* genes induce the SaPI cycle, the induced phage packages and transfers the induced SaPIs at a high rate. In agreement with the previous results, when overexpressed the Dut80α D95E mutant did not increase the transfer of the SaPI islands, confirming its inability to induce the SaPI cycle.

**Table 1:** SaPIbov1 and SaPIbov5 complementation analysis.

Strain	SaPI	Plasmid expressing	SaPI Titre^a,b^	
			No CdCl_2_	1 μM CdCl_2_
JP13746	SaPIbov1	*dut* 80α	8.63 × 10^5^	9.43 × 10^7^
JP13747	SaPIbov1	*dut* 80α D95E	8.07 × 10^2^	3.58 × 10^3^
JP13748	SaPIbov1	—	9.57 × 10^2^	1.47 × 10^3^
JP13749	SaPIbov5	*dut* 80α	7.19 × 10^3^	1.32 × 10^6^
JP13750	SaPIbov5	*dut* 80α D95E	3.68 × 10^3^	4.88 × 10^3^
JP13751	SaPIbov5	—	5.11 × 10^3^	5.09 × 10^3^

^a^Number of transductants per ml of induced culture, using RN4220 as the recipient strain. ^b^The means of the results of three independent experiments are presented. Variation was within 5% in all cases.

### Structure of the Dut80α D95E mutant

**Figure 2:**
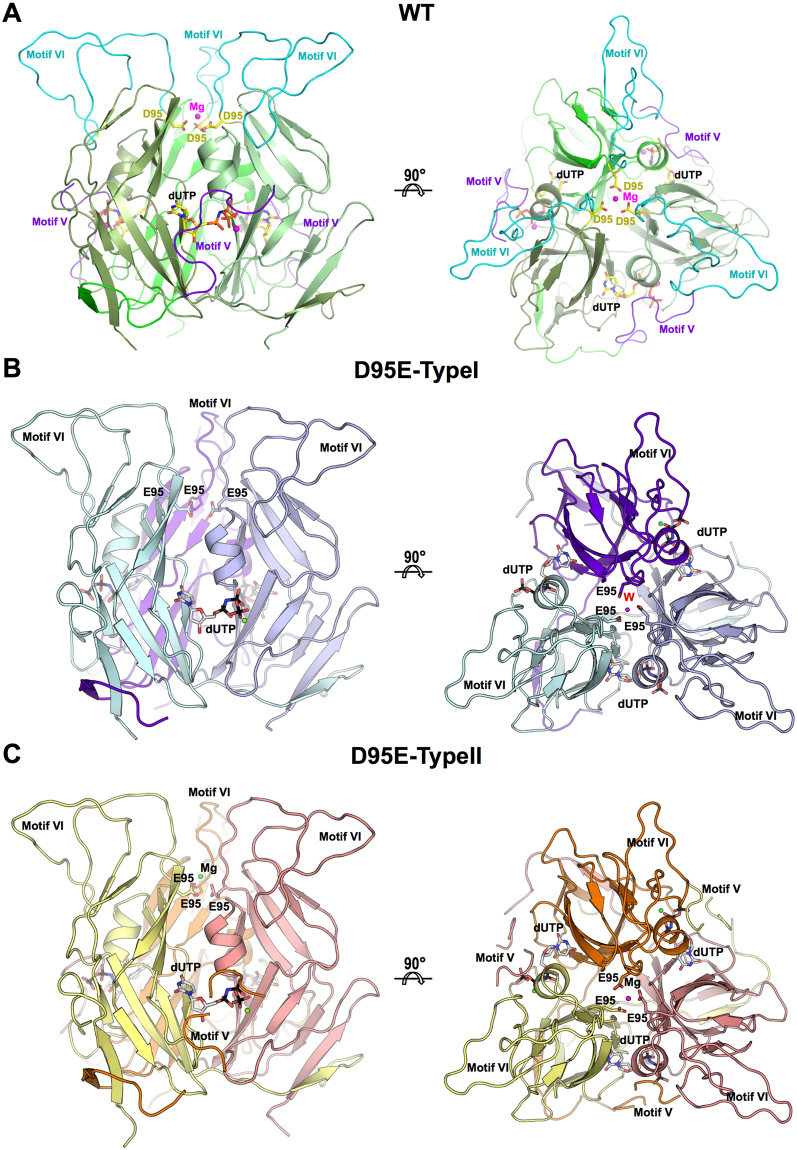
Structures of the Dut80α D95E mutant. Dut80α wt and D95E mutant form similar trimers. Two orthogonal ribbon representations of the trimers from Dut80α (**A**) wt (PDB: 3ZEZ^13^) and D95E (**B**) Type I and (**C**) Type II crystals. Asp95 and Glu95 occupying the central part of trimer channel are represent as sticks in the wt and mutant structures, respectively. The Mg ion coordinated by the acidic residues at position 95 is shown as a sphere and labelled. In the structure of trimeric Dut80α wt (individual monomers in different hues of green) motif V (dark-blue) and motif VI (cyan) involved in Stl interaction are highlighted and labelled. Motif VI are also labelled in D95E Type I (monomers y different hues of blue) and Type II (monomers in yellow, orange and salmon) but Motif V is only labelled in Type II since it is only partially visible in this structure. Nucleotides at the active center are shown as sticks and labelled.

**Figure 3:**
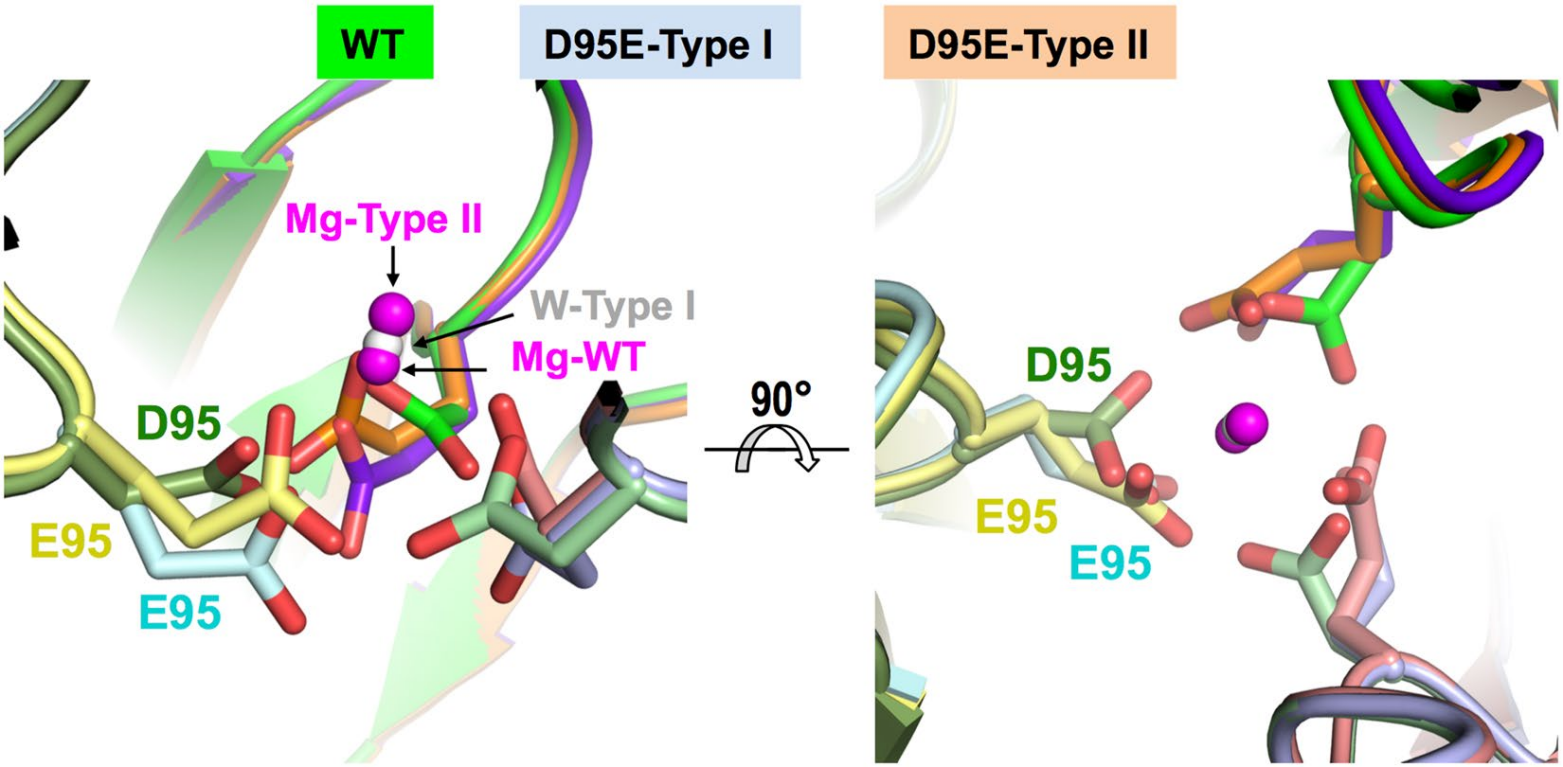
Central channel reorganization induced by D95E mutation. Structural superimposition of trimeric Dut80α wt and D95E structures (coloured as Fig. 2) shows that D95E mutation induces small structural changes in the trimer central channel restricted to the residue 95 side-chain reorientation. Two orthogonal close-views of the central channel around position 95 are shown with these residues as sticks. The disposition of Glu95 in Type I crystal impairs the coordination of the Mg ion in the central channel and this position is occupied by a water molecule (grey sphere labelled as W-Type I). However, in Type II crystals Glu95 side-chains acquire an alternative conformation that allows Mg ion coordination (magenta sphere labelled as Mg-Type II) but displaced with respect to the Mg ion observed in the wt structure (magenta sphere labelled as Mg-WT).

**Figure 4:**
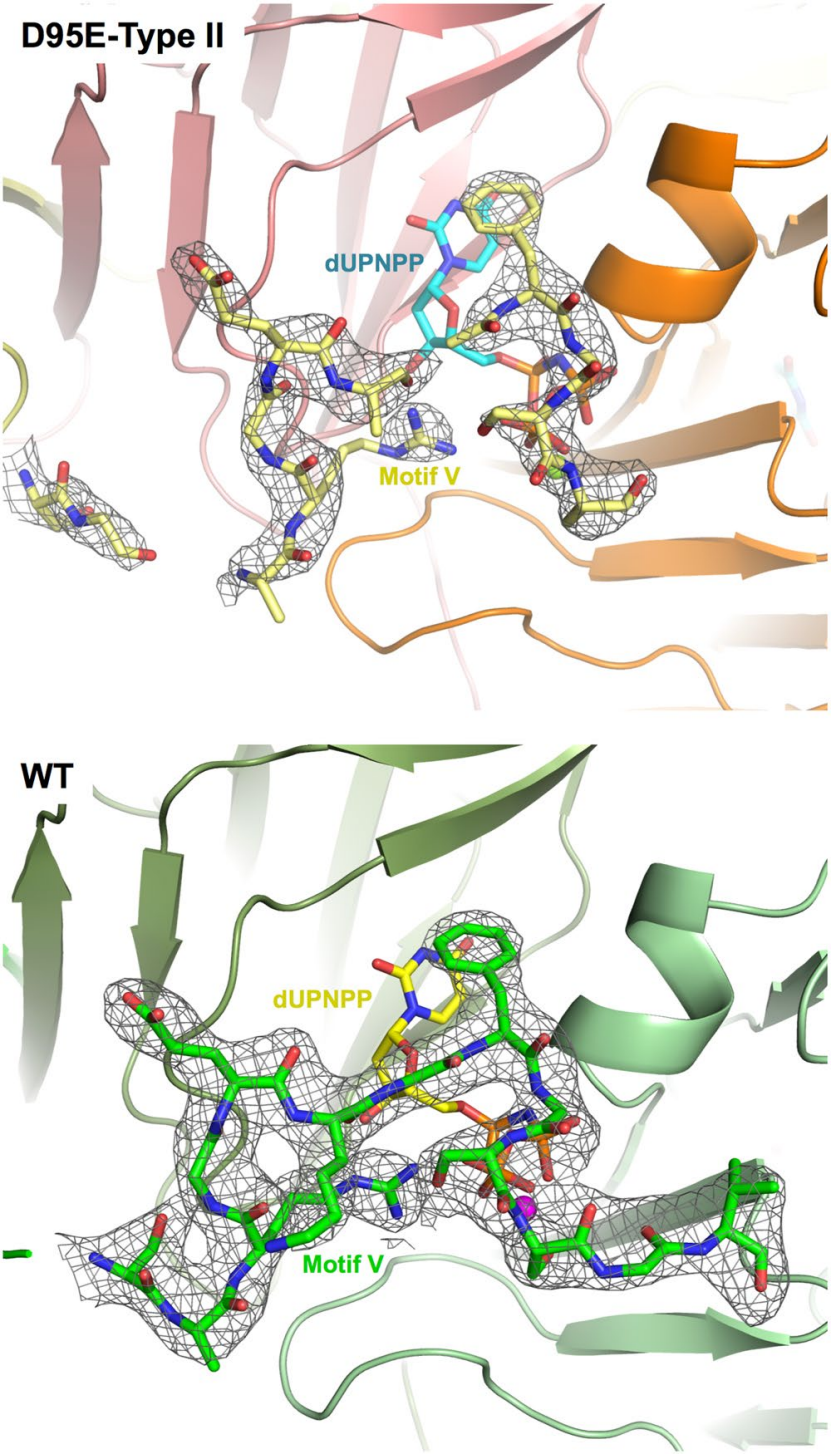
Motif V localization in Dut80α wt and D95E Type II structure. The *2Fo-Fc* maps (contoured at 1.0 σ level) around the C-terminal motif V shown the absence of density for several residues in D95E Type II crystal (top), indicating that this region presents high flexibility. Contrary, the wt structure (PDB: 3ZEZ; bottom) showed a clear density for the complete region^13^. The structures are coloured as in Fig. 2 and residues modelled for motif V are shown in sticks. The nucleotides covered by motif V are shown in stick representation and Mg ions as spheres.

Since Asp95 is placed at the beginning of motif VI (Fig. 2A) and our previous studies had demonstrated that this motif is dispensable for dUTPase activity but is required for SaPI depression^2, 13^, the aforementioned result raised the interesting possibility that the D95E mutation affects the conformation of the specific motif VI. To test this possibility, we solved the X-ray structure of the Dut80α D95E mutant. Asp95 is responsible for chelating a divalent ion in the Dut threefold central channel (Fig. 2A), thus we produced this protein using two alternative protocols: the first of which included MgCl_2_ in the purification buffer, while the second did not, allowing us to evaluate the structural impact of this ion. We obtained crystals in the presence of a non-hydrolyzable dUTP analogue (dUPNPP) for both proteins, which present identical space group and cell dimensions to the previously reported crystals from wt Dut80α protein (Fig. 2B and C, Table 2)^13^. These similarities anticipated almost identical structure. However, the models built from the data obtained with the crystals produced with protein purified in the absence (Type I) or presence (Type II) of Mg ion showed small structural differences but with relevant mechanistic implications. As shown in Fig. 3, the D95E mutation induces the reorientation of the new Glu side-chain in order to avoid the electrostatic repulsion between the carboxylate groups. However, this is accomplished in different ways in both types of crystals. In Type I crystals this is facilitated by a short movement consisting of the side-chain rotation of about 80° around Cβ atom that accommodates the Glu carboxylates deeper in the central channel, however in type II crystals the rotation is much smaller. This is because in Type II crystals a Mg ion occupies an almost identical position at the central part of the channel as was observed in the wt protein, while in Type I crystals the ion position is now occupied by a water molecule (Fig. 3). Remarkably, the Mg ion in Type II crystals maintains a similar coordination sphere as wild-type protein, which is lost in Type I crystals (Fig. 3 and Supplementary Fig. S1). However, this change only induces the motion on different side-chains from neighbour and distant residues, with the main-chain structure of the protein remaining unaltered (RMSD lower than 0.3 Å^2^ for the superposition of all Cβ atoms of both types of crystals with wild-type protein) (Fig. 2 and Supplementary Fig. S2). Contrary to our initial hypothesis, the motif VI remained invariable, although the displacements on this region are higher than protomer average (residues from 114 to 121 showed RMSD > 0.5 Å). Motif VI differences with wild type structure are slightly more obvious at two points: the end (residues 116–121) and the tip (residue 110) of this motif, the latter point being where motifs V and VI are closer (Supplementary Fig. S2). Strikingly, the major structural differences are observed at a distance, suggesting an allosteric action for Mg ion binding in the central channel. In Dut80α D95E type I crystals where the Mg ion is absent, the conserved motif V that covers the active site was totally disordered, even though it was occupied by the nucleotide (Fig. 2B and Supplementary Fig. S2). In contrast, in Type II crystals, which retain the Mg ion, the motif V was visible over the active center with identical conformation to that observed in wild-type protein (Fig. 2), but the density of the map was lower or even absent for some residues (Ser158, Gly164, Gly169 and Val170) (Fig. 4), supporting a weaker stabilization of this loop. In agreement with our previous work in which Dut mutants that were incapable of inducing the SaPI cycle were also affected in their ability to order the conserved motif V, the present structural results confirm the problems that Dut80α D95E has in organizing this motif over the active center even though it is occupied by the nucleotide. This result reinforces the idea that the conserved motif V plays an essential role in the SaPI depression mediated by the phage 80α-coded Dut. Furthermore, Dut80α D95E Type I and II structures confirm the proposed allosteric effect between the threefold central channel and the active center^15^, showing the fine correlation between Mg ion binding and motif V organization. However, it raises the interesting question of how it can be possible that a mutant that has difficulties ordering the conserved motif V retains its enzymatic activity.

**Table 2:** Data Collection and refinement statistics.

Data collection	Dut80α D95E	
	Type I Crystal	Type II Crystal
Beamline	DLS-I.04	ALBA-XALOC
Wavelength (Å)	0.97949	0.97921
Space group	P2_1_3	P2_1_3
Cell dimensions (Å)	a = b = c = 87.19 α = β = γ = 90	a = b = c = 87.27 α = β = γ = 90
Resolution (Å)^*^	38.99–2.85 (3.00–2.85)	87.27–2.5 (2.6 - 2.5)
Unique reflections	5130 (740)	7924 (882)
Completeness (%)	96.1 (96.1)	100 (100)
Multiplicity	2.2 (2.3)	18.0 (18.1)
I/σ(I)	5.2 (2.0)	15.4 (2.0)
Rpim	0.083 (0.320)	0.038 (0.573)
CC(1/2)	0.992 (0.803)	0.898 (0.658)
Refinement		
R_work_	0.2283	0.2257
R_free_	0.2775	0.2805
Number of atoms		
Protein	1200	1300
Ligand/Ions	29	30
Water	21	14
Rmsd, bond (Å)	0.0057	0.0059
Rmsd, angles (°)	1.04	1.14
Ramachandran plot		
Preferred (%)	98.04	95.71
Allowed (%)	1.96	3.68

*Values in parentheses are for highest-resolution shell.

### *In vitro* characterisation of Dut80α D95E mutant

**Figure 5:**
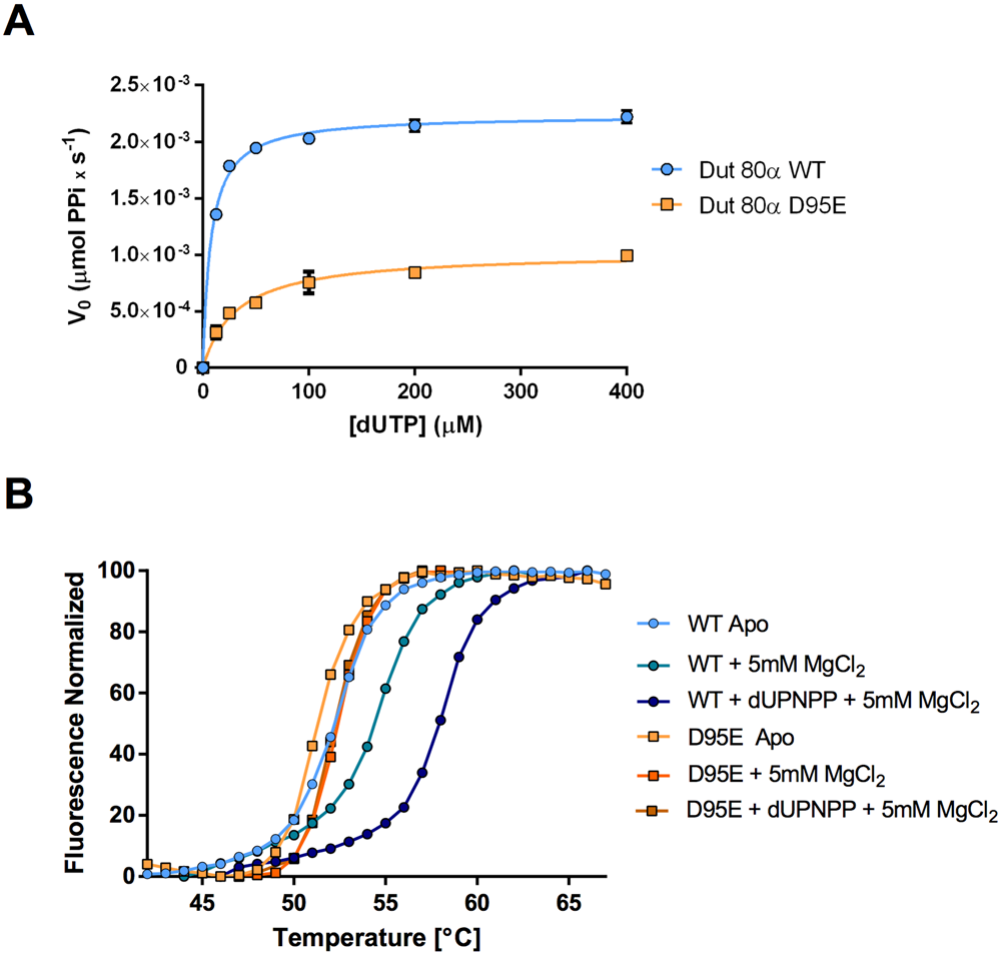
Dut80α D95E mutation decrease protein activity and stability. (**A**) dUTPase activity of Dut80α WT (blue circles) and D95E mutant (orange squares) was measured by malachite green assays. The initial velocities were calculated at different dUTP concentrations and kinetics parameters (Table 3) were determined from these curves using GraphPad software. The curves are the result of six independent assays. (**B**) Thermal-shift assays. Representative thermal denaturation curve profiles of Dut80α wt and D95E mutant in its apo states and in presence of MgCl_2_ or dUPNNP-MgCl_2_. The calculated Tm from at least three independent experiments are shown in Table 3.

The previous results were surprising. On the one hand, our initial studies had suggested that the enzymatic activity was unaffected in this mutant^2^. On the other hand, we had also demonstrated that enzymatic activity was affected in all the previously characterized mutants non-competent in SaPI derepression^13, 17^. Moreover, our recent results suggest that motif V plays a main role as a dUTP-controlled switch in the Stl-Dut interaction. When ordered, motif V impairs access to the Stl binding, interfering with the Stl-Dut interaction. However, motif V is required somehow to stabilise the Stl-Dut complex in the ON state^12^. To solve this mystery, and in order to harmonise these apparently contradictory results, we characterised in depth the kinetic constants of Dut80α D95E mutant. Although Dut80α D95E is catalytically active, as we have previously reported^2^, the analysis revealed that the kinetic parameters Vmax and Km are affected by the mutation (Fig. 5A). As shown in Table 3, Dut80α D95E is slightly less catalytically competent than the wild-type protein with half K_cat_ and around 4 times lower affinity for the dUTP molecule. A similar reduction in K_M_ was observed in the Dut80 motif V deletion mutant^13^, correlating motif V stabilization over the active centre with nucleotide affinity. We further confirmed the K_M_ alteration by ITC, obtaining a reduction in nucleotide affinity of similar magnitude using this complementary approach (Table 3). These results indicate that the mutation produces an allosteric inhibition with uncompetitive phenotype with respect to substrate.

**Table 3:** *In Vitro* characterization of Dut80α WT and D95E.

Protein	Enzyme Kinetics			ITC	Thermal Unfolding (°C)			BLI *K*_D_ (μM)	
	k_cat_ (s^−1^)	K_M_(µM)	k_cat_/K_M_ (M^−1^ s^−1^)	K_D_ (µM)	Apo	+5 mM MgCl_2_	+dUPNPP	Apo	+dUPNPP
Dut80α wt	670,0 ± 6,9	7,5 ± 0,50	89,3 e^6^	2.9	52,1 ± 0,12	54,5 ± 0,12	58 ± 0,09	0,04	0,44
Dut80α D95E	308,7 ± 11,1	32,2 ± 4,24	9,6 e^6^	9.4	51,5 ± 0,02	52,4 ± 0,02	52,3 ± 0,04	BND	BND

BND: Binding not detected in the experimental conditions used. K_D_ > 1 mM.

These results, as reflected in the increased motif V disorder observed in the three-dimensional structures, suggest that the mutation produces a general effect over the protein that epistatically affects the active centre without having a strong local impact on the protein architecture. To confirm this, we analysed the thermal stability of the mutant by thermofluor assay. The wt and mutant forms showed similar thermal denaturation curves with a single step, indicating that trimer dissociation and protomer unfolding are coordinated (Fig. 4B). In agreement with the proposed general destabilization induced by the mutation, a small but consistent reduction in the melting temperature was observed for the Dut80α D95E mutant. Remarkably, the presence of the nucleotide triphosphate increased the melting temperature of the wild type form around six degrees, confirming the close and stable conformation induced by the substrate (Fig. 4C; Table 3). For its part, the Mg ion produced a more modest stabilization of only two degrees (Fig. 4C; Table 3). Conversely, neither the presence of the nucleotide nor the Mg ion had a similar effect on the melting temperature of the Dut80α D95E mutant protein, producing a stabilization lower than one degree (Fig. 4C; Table 3) that correlates closely with the structural and functional data described above. Altogether, these results support the hypothesis that the D95E mutation has a destabilizing effect on the protein that is epistatically transferred over the active centre, disturbing the motif V conformational space and restricting its capacity to adopt a full catalytically competent conformation.

### Analysis of the Stl-Dut D95E interaction

Finally, to complete the characterisation of the Dut80α D95E mutant, we analysed *in vitro* the interaction kinetics of this mutant and the Stl repressor. Previous studies using both phage ϕ11 and 80α have demonstrated the coordinated action of motifs IV, V and VI in Stl recognition and binding, as well as the role of dUTP interference in the interaction^12, 14^. Interaction analyses with Stl using biolayer interferometry (BLI) showed that the D95E mutation completely impairs (K_D_ > 1 mM) the capacity of the Dut80α to interact with the Stl repressor, both in the absence and presence of the nucleotide (Table 3). As shown in Fig. 1, this affinity reduction has dramatic consequences *in vivo*, since the Dut80α D95E cannot induce the SaPI cycle. This behaviour resembles that shown by Dut80α mutants defective in motifs V or VI, as well as the Y84I punctual mutant^12^, indicating that D95E mutation is not restricted to the Stl-Dut complex stabilization mediated by motif V, but is also involved in Stl recognition and binding, which is mediated by motifs IV and VI. As is expected for a general effect, the epistatic action of D95E mutation is not confined to motif V, but also has an impact on motif IV and/or motif VI. This agrees with the structural differences observed between D95E and wild type at the tip of motif VI, just in the region where motifs IV, V and VI converge (Fig. 2A and Supplementary Fig. S2), which we have proposed as the Stl anchor area^12^.

## Discussion

Nucleotide molecules contribute to cell signalling in all forms of life^19^. Among others, cAMP and GTP play pivotal roles by controlling a vast number of cellular pathways in eukaryotes^19^. Alteration of these fine-tuned mechanisms is associated with multiple disease processes, including cancer^20^. Prokaryotic signalling nucleotides such as cyclic di‐AMP (c‐di‐AMP), cyclic di‐GMP (c‐di‐GMP) and guanosine tetra- or pentaphosphate ((p)ppGpp) contribute to bacterial virulence^21–23^. Others and us have proposed that Duts are signalling molecules, involving dUTP as a second messenger^16^. We propose that the Dut proteins carrying extra domains have cognate cellular partners with which they interact using a dUTP-dependent ON/OFF mechanism, thus controlling key cellular processes. Recent exciting evidence using the Stl-Dut model supports this hypothesis. In this model, the dUTP blocks the Stl-Dut interaction, while the degradation of the nucleotide renders the Duts in the competent confirmation capable of inducing the SaPI cycle^12, 14^. Thus, the biological significance of Duts and dUTP has been underestimated to date and the concept of Duts as signalling molecules, involving dUTP as a second messenger, represents a paradigm shift requiring investigation. Dissection of the molecular basis of this novel concept will aid understanding of many biological systems from phage to eukaryotes.

We applied this idea here and continued using the Stl-Dut system to elucidate the mechanism by which the phage Duts perform their regulatory role. To date, all analysis of Dut mutants with altered enzymatic activities has revealed a reduced or null capacity to induce the island^12, 13, 17^. Since all these mutations had affected the active centre of the enzyme, the conserved motif V or the species-specific motif VI, regions than can be directly involved in the binding to the Stl repressor, we decided to analyse in depth the phage 80α Dut D95E mutant. We considered this an interesting mutant because it is enzymatically active, does not induce the island and the mutation is located in a region previously not implicated in the Dut:Stl interaction.

The Asp95 is positioned at the beginning of motif VI, its side-chain directed towards the threefold central channel where, in the trimer, the three carboxyl groups coordinate a metal ion. The presence of metal ions has also been reported in other Duts, mainly those from eukaryotes, although the relative position of the ion within the channel and the coordinating residues are different^15^. For the functionally and structurally related family of DCD-DUT, it has been shown that central channel and active center are connected by a loop, named allosteric loop, at the interface of both structural elements, explaining the allostery observed in these enzymes. Recently, the allosteric behaviour of Duts has been analysed, showing that the active center of these enzymes works independently^15^. The differences in behaviour between DCD-DUT and Dut is explained by the reduction in flexibility of the central channel induced by the presence of the metal ion or/and the introduction of hydrophobic or Pro residues on Duts. This modification not only explains the absence of allosteric communication between active centers but also the increased specificity for dUTP shown by Duts compared to DCD-DUTs. The increment in specificity is further obtained by the concomitant development of the C-terminal P-loop (motif V), characteristic of Duts and absent in DCD-DUTs, that allows the dUDP discrimination and improves the catalytic efficiency of Duts. The evolutive interrelation between the increment of rigidity in the central channel and the emergence of motif V seems to connect both structural elements, as the D95E exemplifies. Our structural data confirms that changes at the central channel that destabilise the trimer are epistatically transferred to the C-terminal motif V, with the structures of Dut80α D95E mutant showing two snapshots of this finely controlled conformational process. Furthermore, we have shown that in the case of phage-encoded Duts, such changes also affect other structural elements such as the tip of the motif VI. In the latter case, we cannot distinguish whether it is a direct effect, since Asp95 is placed at the beginning of motif VI, or an indirect effect via motif V, since our previous structural data showed that both elements interact. Remarkably, these small epistatic effects in different structural elements are not sufficient to abolish catalytic activity but have an enormous impact on the capacity of the Dut80α to interact with the Stl repressor. This effect is explained by the synergistic contribution of three different structural elements (motifs IV, V and VI) in the Stl-binding process^12^. We anticipate here that these results, based on the analysis of the Dut:Stl interaction, will have enormous relevance in our understanding of how the Dut enzymes perform their regulatory roles. As previously mentioned, we propose that Duts are signalling molecules, from phage to humans. Since we are at the beginning of a very long journey, it is obvious that we do not know yet the cellular pathways controlled by these enzymes. However, we consider that the Stl:Dut interaction provides an interesting model with which to address some of these interesting scientific questions.

## Methods

### Bacterial strains and growth conditions

The bacterial strains used in these studies are listed in Supplementary Table S1. The procedures for preparation and analysis of phage lysates, in addition to transduction and transformation of *S. aureus*, were performed essentially as previously described^10, 24^.

### DNA methods

General DNA manipulations were performed using standard procedures. The oligonucleotides used in this study are listed in Supplementary Table S2. The labelling of the probes and DNA hybridization were performed according to the protocol supplied with the PCR-DIG DNA-labelling and Chemiluminescent Detection Kit (Roche).

### Plasmid construction

The plasmid constructs expressing the different Dut proteins were reported previously or were prepared by cloning PCR products obtained with the oligonucleotide primers listed in Supplementary Table S2. Dut proteins were expressed in *S. aureus* under inducing conditions from the P*cad* promoter in the expression vector pCN51, as previously described^13^.

### Protein expression and purification

Dut80α wild type and Dut80α D95E mutant cloned into pET28a (pET28a-Dut80α WT and pET28a-Dut80α D95E) expression vector were transformed into *E. coli* BL21 (DE3) strain form Novagen. The over-expression of both proteins was done as previously described^13^. Briefly, proteins were over-expressed by first growing the cells to exponential phase (optical density of O.D. = 0.6 at measured at λ = 600 nm) at 20 °C in LB medium supplemented with 33 g/ml kanamycin, followed by the addition of 1 mM Isopropyl-β-D-1-thiogalactopyranoside (IPTG) for 16 h.

After the induction, cells over-expressing proteins were harvested by centrifugation, and two alternative purification protocols were used. The first protocol was used to produce protein in magnesium-free conditions (Type I). In this protocol, the cells were resuspended in Mg-free buffer A (75 mM HEPES pH 7.5, 500 mM NaCl) supplemented with 1 mM phenylmethanesulfonyl fluoride (PMSF) and lysed by sonication. The lysate was clarified by centrifugation and the soluble fraction was loaded on a His Trap HP column (GE Healthcare) pre-equilibrated with Mg-free buffer A. The column was washed with the same buffer supplemented with 10 mM imidazole and proteins were eluted with Mg-free buffer A supplemented with 500 mM imidazole. The eluted proteins were concentrated and loaded onto a Superdex S200 (GE Healthcare) equilibrated with Mg-free buffer B (75 mM HEPES pH 7.5, 250 mM NaCl) for size exclusion chromatography. The fractions were analyzed by SDS/PAGE and those fractions showing purest protein were selected, concentrated and stored at −80 °C. The second protocol was used to purify Dut80α D95E mutant in the presence of Mg (Type II). This protocol is almost identical to the previous one but the buffer C (75 mM HEPES pH 7.5, 400 mM NaCl and 5 mM MgCl_2_) was used in all the chromatographic steps.

Stl from SaPIbov1 was produced in *E. coli* BL21 (DE3) (Novagen) strain and purified as previously described^12^.

### dUTPase activity assay

Malachite Green phosphate assay was used to measure the dUTPase activity as previously described^12^. Briefly, Pi released was quantified in 200 μl assay volume of reaction buffer containing 75 mM HEPES pH 7.5, 250 mM NaCl, 5 mM MgCl_2_ and 0.01 U of inorganic pyrophosphatase (Thermo scientific), along with 0.075 μg of the corresponding Dut. The reactions were started by addition of dUTP at different concentrations (12.5, 25, 50, 100, 200 and 400 μM final concentration) and stopped samples were taken at 0, 2, 4, 6, 8 and 10 min by adding 50 μl of malachite green development solution to stop the reaction. After 10 min incubation at room temperature, the Pi production was calculated based on the absorbance at 630 nm and against a previously determined standard curve for Pi. Reactions showed linearity over the time-course of the reaction and the initial velocity was calculated following this procedure using GraphPad Prism software.

### Thermal shift assay

The thermal shift assay was conducted in the 7500 Real-Time PCR System (Applied Biosystems). Samples of 20 μl containing 5× Sypro Orange (Sigma) and 10 μM of protein in a buffer containing 75 mM HEPES pH 7.5 and 250 mM NaCl were loaded in 96-well PCR plates. When indicated, the sample mix was supplemented with 5 mM MgCl_2_ or 1 mM of 2′-Deoxyuridine-5′-[(α,β)-imido]triphosphate (dUPNPP; Jena Bioscience) plus 5 mM MgCl_2_. Samples were heated from 20 to 85 °C with a heating rate of 1 °C/min. The fluorescence intensity was normalized and analysed using GraphPad Prism software.

### Biolayer Interferometry (BLI)

The binding affinity (K_D_) between Duts and Stl was measured by Biolayer Interferometry (BLI) using the BLITz system (FortéBio) as previously described^12^. When the influence of nucleotide in the binding process was analysed, the reaction buffers were supplemented with 0.5 mM dUPNPP. For each interaction, the corresponding His-tagged Dut was immobilized on Ni-NTA biosensors (FortéBio) at 1 μM concentration. At least five different dilutions of Stl (from 0.062 to 4 μM plus the reference without Stl) were used in the assays, adjusting the highest concentration of Stl to 10 times the estimated K_D_ (Table 3). Binding affinity calculation and data analysis were performed with BLItz Pro 1.2 software. A 1:1 model was employed to fit the data.

### Dut80α D95E crystallization, data collection and structure determination

Dut80α D95E proteins were crystallized at 21 °C using sitting drop method in the Crystallogenesis facility of IBV. To obtain Dut80α D95E crystals in complex with dUPNPP the protein (5–10 mg/ml) was mixed with 1 mM dUPNPP and 5 mM MgCl_2_ prior the screening. Final crystallization conditions for Dut80α D95E purified in absence of Mg (Type I) contained 32% Ethanol, 2% PEG 1000 and 0.1 M of phosphate citrate pH 4.2. For Dut80α D95E purified in presence of Mg (Type II) better crystals were obtained in conditions containing 20% Ethanol and 10% Glicerol. Type II crystals were cryo-protected in liquid N_2_ using crystallization conditions and Type I crystals were cryo-protected by increasing ethanol to 40%. X-ray diffraction was performed at 100 K in Diamond Light Source (DLS) and ALBA synchrotrons.

Processing of collected data collection was performed with XDS^25^ and iMosflm program^26^. Statistics for data processed are shown in Table 2. Structures were solved at 2.85 and 2.5 Å resolution for Type I and Type II crystals, respectively. For both types of crystals, structure determination was made by molecular replacement using Phaser program^27^ and an edited Dut80α PDB model (PDB 3ZEZ). Based on previously reported results^13^, we excluded from the starting model the high flexibility motif V and the non-conserved motif VI in trimeric Duts (amino acids range 142–170 and 95–127, respectively). This decision was made in order to reduce the imposition of any initial structural conformation to the flexible motif V and the non-conserved motif VI avoiding a possible bias of structural data. Iterative refinement, rebuilding and validation steps were done using programs Coot^28^ and Phenix^29^. Final model for Type I crystal includes one Dut80α D95E molecule (residues 2–156) with one dUPNPP-Mg bound at the active center. Final model of Type II crystal includes one Dut80α D95E molecule (residues 2–157 plus 159–168), one dUPNPP-Mg bound at the active center and one Mg ion bound at the central channel. Refinement statistics and models composition are shown in Table 2. Stereochemical properties were assessed by wwwPDB X-ray Validation server (https://validate-rcsb-1.wwpdb.org).

### Data availability statement

All data generated or analysed during this study are included in this published article (and its Supplementary Information files). The X-ray structures (coordinates and structure factors) of Dut80α D95E Type I and Type II crystals have been submitted to Protein Data Bank under accession codes 5NYZ and 5NZ2, respectively.

## Electronic supplementary material

Supplementary information
