# Supplementary material for: Dissecting the link between the enzymatic activity and the SaPI inducing capacity of the phage 80α dUTPase
Source: Sci Rep. 2017 Sep 11;7:11234. doi: 10.1038/s41598-017-11234-9 (PMC5593958; doi:10.1038/s41598-017-11234-9)
Supplement: Supplementary file 1 — Supplementary information [file 41598_2017_11234_MOESM1_ESM.pdf]

Supplementary Material

Dissecting the link between the enzymatic activity and the SaPI inducing capacity of the phage 80α dUTPase.

Christian Alite[a,$], Suzanne Humphrey [b,$], Jordi Donderis[a,$] Elisa Maiques[a], J. Rafael Ciges-Tomas[a], José R. Penadés[b,*], Alberto Marina[a,*]

[a]Instituto de Biomedicina de Valencia (IBV-CSIC) and CIBER de Enfermedades Raras (CIBERER), Jaume Roig 11, 46010-Valencia, Spain; [b]Institute of Infection, Immunity and Inflammation, College of Medical, Veterinary and Life Sciences, University of Glasgow, Glasgow, G12 8TA, UK

[$] Equal Contribution

Supplementary Figures 1-3
Supplementary Tables 1-2

* Correspondence: Alberto Marina
Instituto de Biomedicina de Valencia (IBV-CSIC)
Jaume Roig 11
46010-Valencia, Spain
e-mail: amarina@ibv.csic.esamarina@ibv.csic.es
Phone: +34-963391754
Fax: +34-963690800

José R. Penadés
Institute of Infection, Immunity and Inflammation
120 University Place
Glasgow G12 8TA; UK
e-mail: JoseR.Penades@glasgow.ac.uk
Phone: +44 (0) 141 330 8770
Fax:

Supplementary Figures

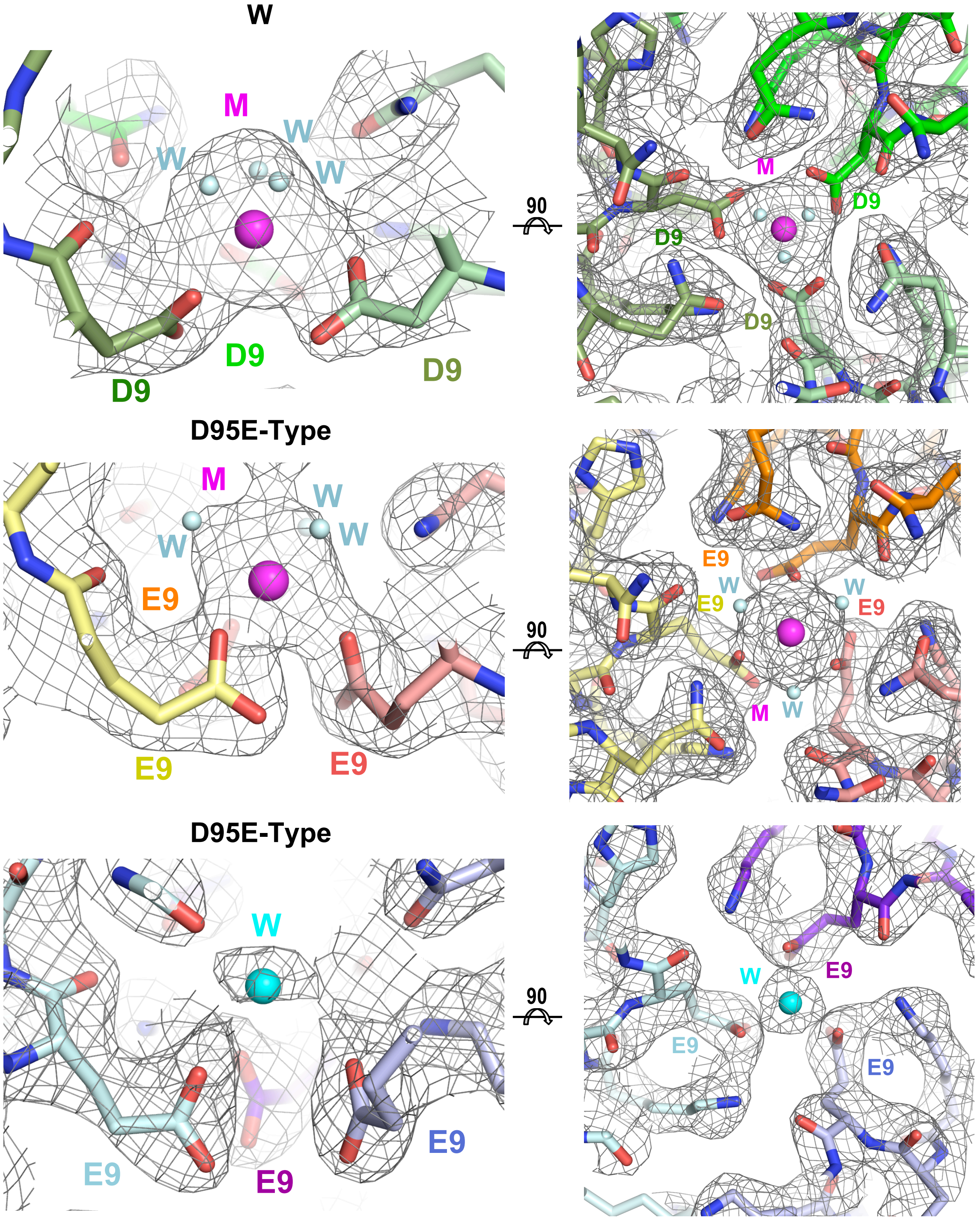

Fig. S1. Residue 95 localization in Dut80α WT and D95E mutant structures. Close views of the central channel around the residue 95 in the Dut80α WT (PDB: 3zEZ; [1]) and D95E Type I and Type II crystals showing the 2Fo–Fc maps used to model the structures (contoured at 1.0 σ level). The residues are shown in sticks and the position 95 is labeled. The Mg ion and its coordination water molecules can be modeled in WT and D95E Type II maps. In the case of D95E Type I crystal only a water molecule could be modeled at this position.

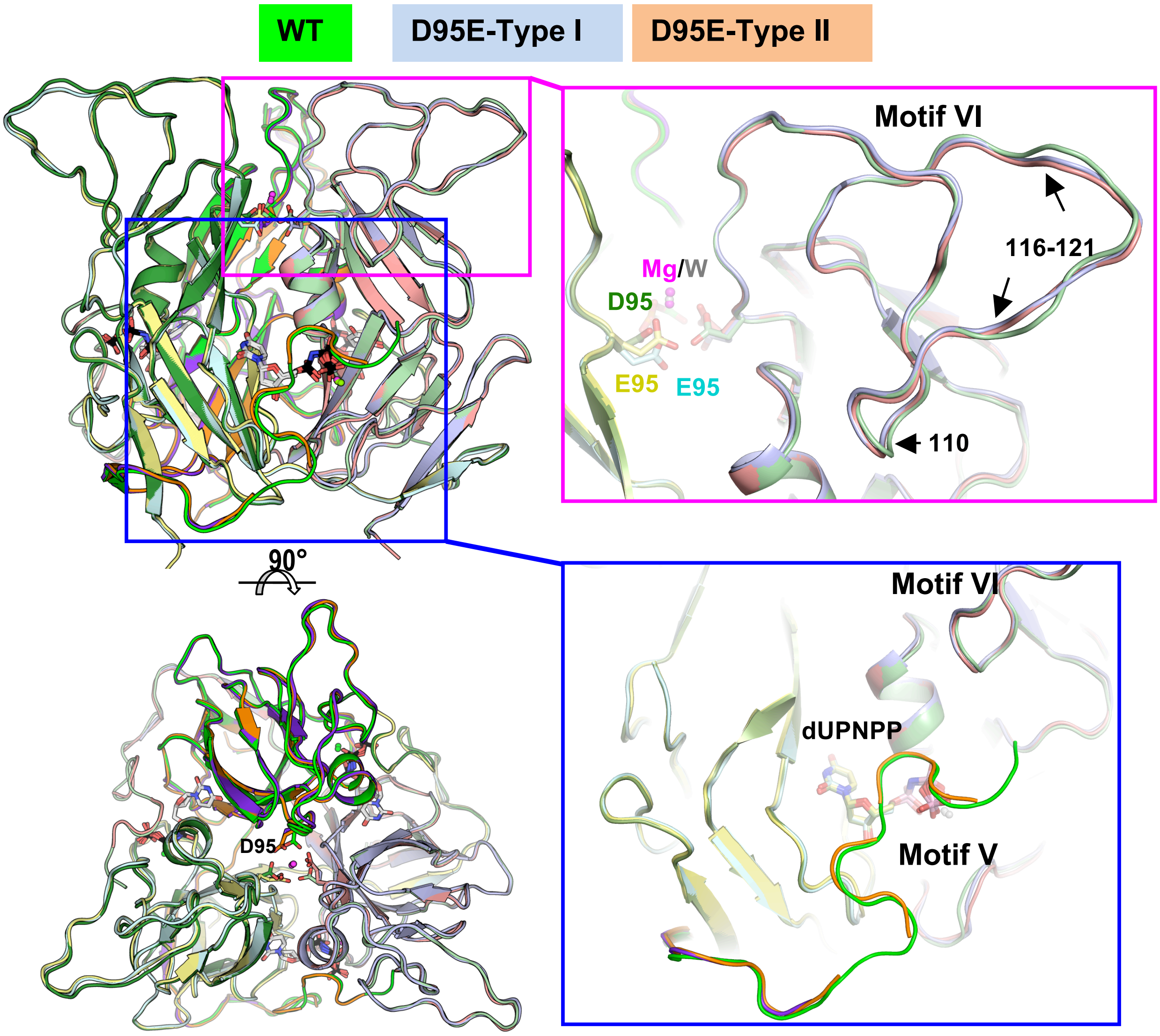

Fig. S2. Structural comparison of Dut80α WT and D95E mutant structures The structural superimposition of trimeric Dut80α WT (green hues) and D95E mutant Type I (blue hues) and Type II (orange hues) are shown in two orthogonal views (left). The three structures are virtually identical except in the motif VI region (right-up) where small displacements are observed, and in the Motif V region (right bottom) that acquire mobility in D95E that precludes its partial (Type II) or total (Type I) localization. Residue at position 95 and nucleotides are shown is sticks and labeled.

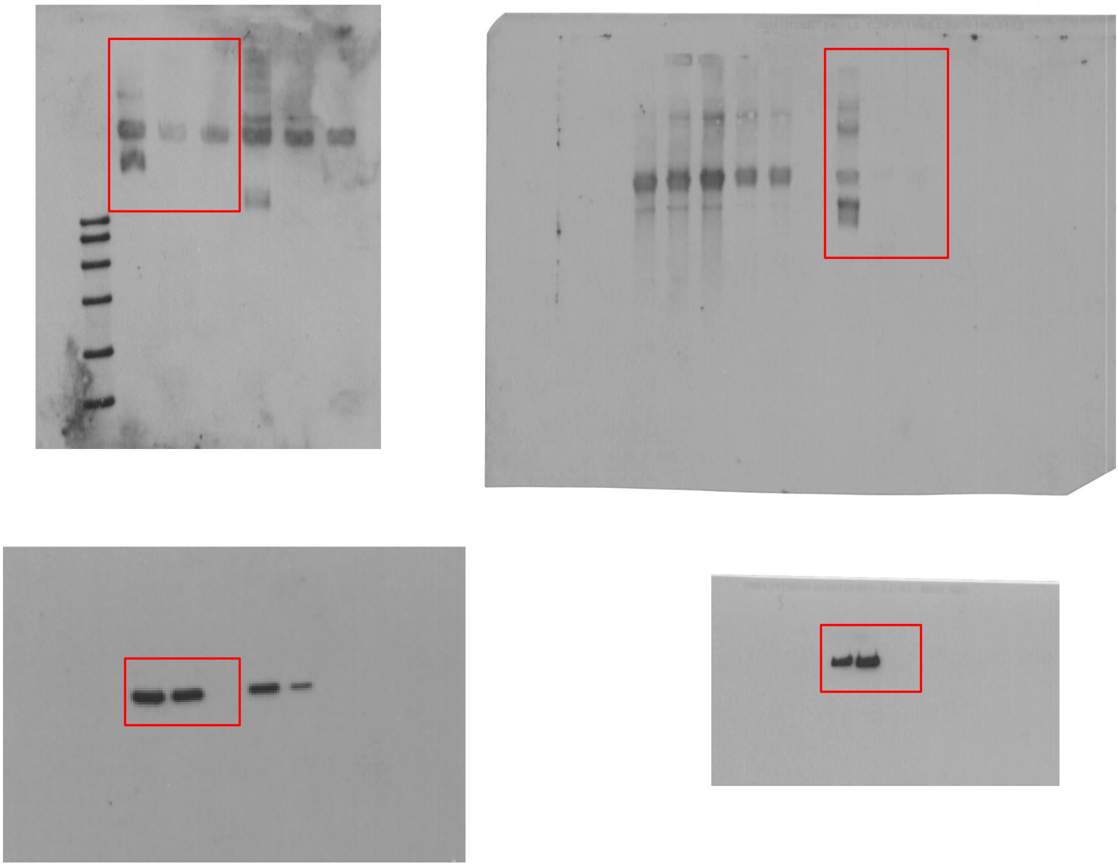

Fig. S3. Full-length blots for Figure 1A. The blot portions showed in Figure 1A are highlighted with a red box.

Supplementary Tables

Table S1: Strains and plasmids used in this study

Strains	Description	Reference
RN4220	Restriction-defective derivative of RN450	[2]
RN450	NCTC8325 cured of ϕ11, ϕ12 and ϕ13	[3]
RN10359	RN450 80α	[4]
JP6681	RN4220 Δ*spa*	[5]
JP4480	RN10359 ϕ80α Δ*dut*	[5]
JP13753	JP6681 pJP821	This work
JP13729	JP6681 pJP1785	This work
JP13756	JP6681 pCN51	This work
JP6774	JP6681 SaPIbov1 *tst::tetM*	[5]
JP11651	JP6774 pJP821	[5]
JP13733	JP6774 pJP1785	This work
JP13755	JP6774 pCN51	This work
JP13731	JP6681 SaPIbov5 *vwb::tetM*	This work
JP13744	JP13731 pJP821	This work
JP13745	JP13731 pJP1785	This work
JP13754	JP13731 pCN51	This work
JP12491	JP6681 pJP674	[5]
JP9033	JP6681 pJP674 pJP821	[5]
JP13730	JP6681 pJP674 pJP1785	This work
JP4496	JP4480 SaPIbov1 *tst::tetM*	[5]
JP13746	JP4496 pJP821	This work
JP13747	JP4496 pJP1785	This work
JP13748	JP4496 pCN51	This work
JP13732	JP4480 SaPIbov5 *vwb::tetM*	This work
JP13749	JP13732 pJP821	This work
JP13750	JP13732 pJP1785	This work
JP13751	JP13732 pCN51	This work

Plasmids	Description	Reference
pCN51	Expression vector	[6]
pJP674	pRN8298-clor-pInt-20-19-18*blaZ*	[5]
pJP821	pCN51-3xFLAG-*dut*80α	[5]
pJP1785	pCN51-3xFLAG-*dut*80α D95E	This work

Table S2: Oligonucleotides used in this study

Plasmid	Oligonucleotides	Sequence (5’-3’)
pJP1785	orf32phi80alpha-16mS	ACGCGTCGACATTATGGCAGGTCAAGTTGTC
	dutphi80alpha-12m	GGGATTAATATCAAGAATGAGCATGAAGATGACAAAATGC
	dutphi80alpha-13c	GCATTTTGTCATCTTCATGCTCATTCTTGATATTAATCCC
	orf32phi80alpha-2cB	CGCGGATCCTCACCAAAACCTCCTTGACTC
Probe	Oligonucleotides	Sequence (5’-3’)
SaPIbov1	SaPIbov1-112mE	CCGGAATTCAATTGCTGAGGCAAAACTTC
	SaPIbov1-113cB	CGCGGATCCTAATTCTCCACGTCTAAAGC

Restriction sequences are underlined.

References

[1] M.Á. Tormo-Más, J. Donderis, M. García-Caballer, A. Alt, I. Mir-Sanchis, A. Marina, et al., Phage dUTPases control transfer of virulence genes by a proto-oncogenic G protein-like mechanism, Mol. Cell. 49 (2013) 947–958. doi:10.1016/j.molcel.2012.12.013.

[2] B.N. Kreiswirth, S. Löfdahl, M.J. Betley, M. O'Reilly, P.M. Schlievert, M.S. Bergdoll, et al., The toxic shock syndrome exotoxin structural gene is not detectably transmitted by a prophage, Nature. 305 (1983) 709–712.

[3] R. Novick, Properties of a cryptic high-frequency transducing phage in *Staphylococcus aureus*, Virology. 33 (1967) 155–166.

[4] C. Ubeda, P. Barry, J.R. Penadés, R.P. Novick, A pathogenicity island replicon in *Staphylococcus aureus* replicates as an unstable plasmid, Proc. Natl. Acad. Sci. U.S.a. 104 (2007) 14182–14188. doi:10.1073/pnas.0705994104.

[5] M.Á. Tormo-Más, I. Mir, A. Shrestha, S.M. Tallent, S. Campoy, I. Lasa, et al., Moonlighting bacteriophage proteins derepress staphylococcal pathogenicity islands, Nature. 465 (2010) 779–782. doi:10.1038/nature09065.

[6] E. Charpentier, A.I. Anton, P. Barry, B. Alfonso, Y. Fang, R.P. Novick, Novel cassette-based shuttle vector system for gram-positive bacteria, Appl. Environ. Microbiol. 70 (2004) 6076–6085. doi:10.1128/AEM.70.10.6076-6085.2004.
